# Clip placement to prevent delayed bleeding after colonic endoscopic mucosal resection (CLIPPER): study protocol for a randomized controlled trial

**DOI:** 10.1186/s13063-020-04996-7

**Published:** 2021-01-18

**Authors:** Ayla S. Turan, Leon M. G. Moons, Ramon-Michel Schreuder, Erik J. Schoon, Jochim S. Terhaar sive Droste, Ruud W. M. Schrauwen, Jan Willem Straathof, Barbara A. J. Bastiaansen, Matthijs P. Schwartz, Wouter L. Hazen, Alaa Alkhalaf, Daud Allajar, Muhammed Hadithi, Bas W. van der Spek, Dimitri G. D. N. Heine, Adriaan C. I. T. L. Tan, Wilmar de Graaf, Jurjen J. Boonstra, Fia J. Voogd, Robert Roomer, Rogier J. J. de Ridder, Wietske Kievit, Peter D. Siersema, Paul Didden, Erwin J. M. van Geenen, on behalf of the Dutch EMR Study Group

**Affiliations:** 1 Department of Gastroenterology and Hepatology, Radboud University Medical Center, Radboud Institute of Health Sciences, Nijmegen, Netherlands; 2 Department of Gastroenterology and Hepatology, University Medical Center Utrecht, Utrecht, Netherlands; 3 Department of Gastroenterology and Hepatology, Catharina Hospital, Eindhoven, Netherlands; 4 Department of Gastroenterology and Hepatology, Jeroen Bosch Hospital, s’ Hertogenbosch, Netherlands; 5 Department of Gastroenterology and Hepatology, Bernhoven, Uden, Netherlands; 6 Department of Gastroenterology and Hepatology, Màxima Medical Center, Veldhoven, Netherlands; 7 Department of Gastroenterology and Hepatology, Amsterdam University Medical Centers, location Academic Medical Center, Amsterdam, Netherlands; 8 Department of Gastroenterology and Hepatology, Meander Medical Center, Amersfoort, Netherlands; 9 Department of Gastroenterology and Hepatology, Elisabeth-Tweesteden Hospital, Tilburg, Netherlands; 10 Department of Gastroenterology and Hepatology, Isala Clinics, Zwolle, Netherlands; 11 Department of Gastroenterology and Hepatology, Hospital St. Jansdal, Harderwijk, Netherlands; 12 Department of Gastroenterology and Hepatology, Maasstad Hospital, Rotterdam, Netherlands; 13 Department of Gastroenterology and Hepatology, Noordwest Hospital Group, Alkmaar, Netherlands; 14 Department of Gastroenterology and Hepatology, Canisius-Wilhelmina hospital, Nijmegen, Netherlands; 15 Department of Gastroenterology and Hepatology, Erasmus Medical Center, Rotterdam, Netherlands; 16 Department of Gastroenterology and Hepatology, Leids University Medical Center, Leiden, Netherlands; 17 Department of Gastroenterology and Hepatology, Medical Center Leeuwarden, Leeuwarden, Netherlands; 18 Department of Gastroenterology and Hepatology, Franciscus Gasthuis, Rotterdam, Netherlands; 19 Department of Gastroenterology and Hepatology, Maastricht University Medical Center+, Maastricht, Netherlands; 20 IQ Healthcare, Radboud University Medical Center, Nijmegen, Netherlands

**Keywords:** Prophylactic clipping, EMR, Colonic polyp, Delayed bleeding, Clip artifact

## Abstract

**Background:**

Endoscopic mucosal resection (EMR) for large colorectal polyps is in most cases the preferred treatment to prevent progression to colorectal carcinoma. The most common complication after EMR is delayed bleeding, occurring in 7% overall and in approximately 10% of polyps ≥ 2 cm in the proximal colon. Previous research has suggested that prophylactic clipping of the mucosal defect after EMR may reduce the incidence of delayed bleeding in polyps with a high bleeding risk.

**Methods:**

The CLIPPER trial is a multicenter, parallel-group, single blinded, randomized controlled superiority study. A total of 356 patients undergoing EMR for large (≥ 2 cm) non-pedunculated polyps in the proximal colon will be included and randomized to the clip group or the control group. Prophylactic clipping will be performed in the intervention group to close the resection defect after the EMR with a distance of < 1 cm between the clips. Primary outcome is delayed bleeding within 30 days after EMR. Secondary outcomes are recurrent or residual polyps and clip artifacts during surveillance colonoscopy after 6 months, as well as cost-effectiveness of prophylactic clipping and severity of delayed bleeding.

**Discussion:**

The CLIPPER trial is a pragmatic study performed in the Netherlands and is powered to determine the real-time efficacy and cost-effectiveness of prophylactic clipping after EMR of proximal colon polyps ≥ 2 cm in the Netherlands. This study will also generate new data on the achievability of complete closure and the effects of clip placement on scar surveillance after EMR, in order to further promote the debate on the role of prophylactic clipping in everyday clinical practice.

**Trial registration:**

ClinicalTrials.gov NCT03309683. Registered on 13 October 2017. Start recruitment: 05 March 2018. Planned completion of recruitment: 31 August 2021.

**Supplementary Information:**

The online version contains supplementary material available at 10.1186/s13063-020-04996-7.

## Background

### Delayed bleeding after endoscopic mucosal resection

In 2014, a national colorectal cancer screening (NCCS) program was introduced in the Netherlands [1]. The program is based on immunological fecal occult blood testing (iFOBT), followed by colonoscopy after a positive iFOBT result. During colonoscopy, the detection of colorectal cancer and advanced adenomas has been found to be 8% and 43%, respectively [2]. The treatment of advanced adenomas has resulted in a considerable spin-off of the NCCS program.

Endoscopic mucosal resection (EMR) is a safe and cost-effective method for resecting larger flat or sessile adenomas in the colorectum with no signs of submucosal invasion. However, delayed bleeding (DB) is the most prevalent complication and is reported in up to 12% after EMR. Identified risk factors of DB after EMR are anticoagulant drug use within 7 days of the procedure (OR 6.3; *P* = 0.005), polyp size and location in the colon with a 12% incidence rate of delayed bleeding in the cecum, 10% in the proximal ascending colon, 7% at the hepatic flexure, and 2–3% in the left colon [3–9].

Several preventive measures have been undertaken to reduce post-EMR bleeding, such as coagulation of visible vessels, prophylactic clipping (PC), or suturing of the EMR resection defect. Prophylactic coagulation of visible vessels in the resection defect has not been shown to decrease the incidence of DB [7]. However, PC has been reported in several studies to reduce DB especially in right sided EMR’s for lesions sized over 2 cm [10, 11]. Theoretically, a clip applies pressure to the underlying vessels in the EMR defect and results in increased mucosal healing [12], which may result in a reduced DB risk. Nonetheless, studies reporting on PC in EMR have several limitations, such as a retrospective design, inclusion of all sizes and types of polyps (pedunculated/flat, right/left-sided), lack of statistical power, and all reported studies were performed in tertiary referral centers, thereby not representing normal daily practice. Additionally, PC will lead to increased costs of EMR and it is unknown whether the additional costs of PC in high-risk patients (right sided flat polyps ≥ 2 cm) will outweigh the benefits of PC in terms of quality of life gains and/or cost savings related to prevention of DB [12].

### Scar surveillance

After piecemeal EMR, guidelines recommend surveillance colonoscopy after 6 months to evaluate the presence of residual adenoma [13]. Nonetheless, examining post-EMR scars with white-light endoscopy alone may miss up to 30% of recurrences revealed by random biopsy [14, 15]. Thorough inspection of the scar area with enhanced imaging (e.g. NBI, I-scan, etc.) is one of the techniques to determine the presence of recurrence.

With PC, the apposition of the defect margins with clips may cause a different appearance of the scar leading to difficulty in assessing a potential recurrence. This clip-induced scar pattern occurs in 30% of the EMR sites and is described as a “clip artifact”: a bumpy scar that has a normal pit pattern and is normal on biopsy [14, 15]. The occurrence of clip artifacts may therefore increase the difficulty of detecting recurrences.

### Aims

We designed a nationwide randomized controlled trial aiming to compare PC after EMR to standard EMR care without PC for the prevention of clinically significant DB < 30 days. In addition, we aimed to determine DB severity, rates of recurrent and residual adenoma and clip artifacts after PC, and cost-effectiveness compared with standard care.

## Methods

### Trial design

The CLIPPER trial is a nationwide multicenter randomized, parallel-group, patient-blinded superiority trial, comparing prophylactic clipping after EMR to standard care in 356 patients undergoing EMR for a non-pedunculated polyp in the proximal colon sized 20–60 mm in 19 hospitals of the Dutch EMR Study Group in the Netherlands. The trial will be conducted over a time period of 3 years. The study protocol was written in accordance with the SPIRIT guidelines [16, 17].

### Eligibility criteria

Patients aged ≥ 18 years undergoing EMR of a flat or sessile colonic polyp (Paris classification 0-IIa/b/c, Is) measuring 20–60 mm and located proximal from the splenic flexure who gave written informed consent prior to EMR are eligible for inclusion in the study.

A subject with any of the following exclusion criteria prior to randomization will be excluded from participation in this study:

- Pregnancy
- Active inflammatory colonic conditions (e.g., inflammatory bowel disease)
- American Society of Anesthesiology (ASA) grades IV–V
- Previous resection or attempted resection of a lesion less than 30 days ago
- EMR for residual adenoma still in place after a previous intervention
- > 1 lesion to be removed in the same session
- Involvement of the cecal valve or orificium of the appendix
- Endoscopic appearance of invasive malignancy (non-lifting Kato D, Kudo V pit pattern)
- Macroscopic non-radical resection
- Clip deployed prior to the completion of the EMR for a perforation or a major intra-procedural bleeding that cannot be treated with coagulation

### Trial treatment

#### Standard care: no PC

Applying snare tip soft coagulation to the margins of the post-EMR defect is often used as preventive treatment for recurrence and may be applied at the endoscopists’ discretion. Anticoagulant use will be managed according to the Dutch Society of Gastroenterologists guideline 2016 [18].

#### Intervention: PC

**Fig. 1:**
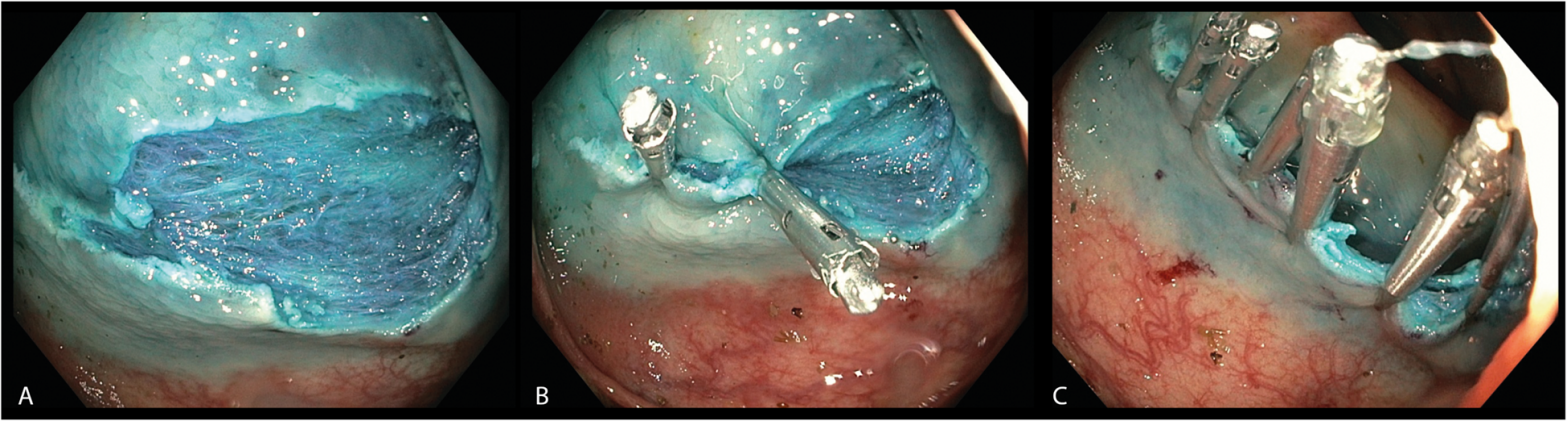
Prophylactic clip closure in a zipper fashion. **a** A mucosal defect after EMR. In **b**, two clips are placed in a zipper fashion, approximating the defect margins. In **c**, clipping is complete after six clips have been placed in a zipper fashion

**Fig. 2:**
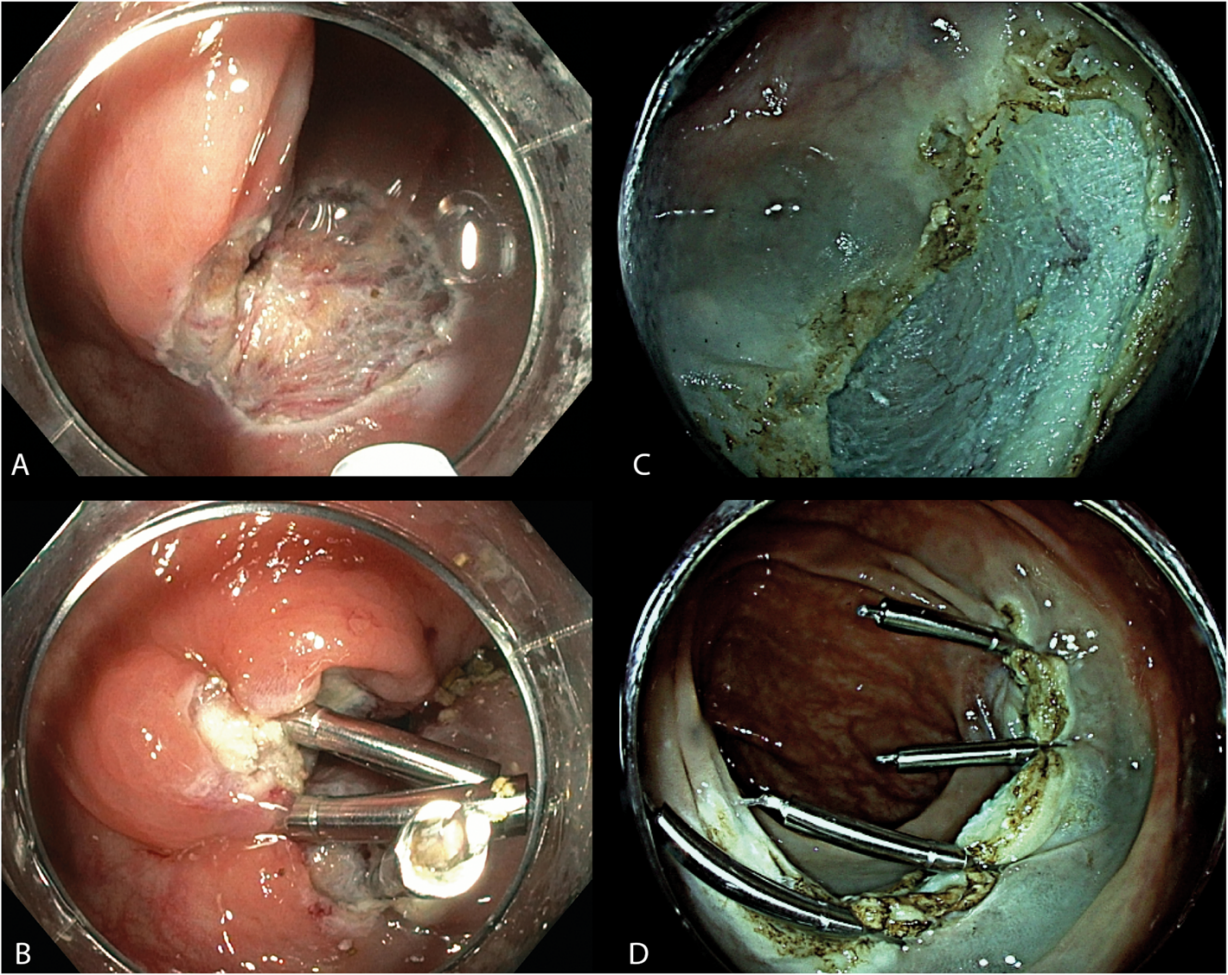
EMR defects with prophylactic clip closure. **a** A mucosal defect after EMR. In **b**, the EMR defect from A has been approximated with three clips. **c** Another mucosal defect after EMR. In **d**, the EMR defect from **c** has been closed with four clips

PC is standardized using Quick Clip Pro - Single Use Repositionable Clips (Olympus, Japan) and is performed in a zipper fashion (Fig. 1). Successful PC is defined as complete closure of the resection defect with aligning clips placed 0.5–1.0 cm apart (Figs. 1 and 2) [10, 19]. After PC, a picture of the final result is made.

All patients will receive normal standard of care in terms of day of discharge, instructions at discharge, outpatient clinic visits to discuss pathology reports, and additional treatment.

#### Surveillance

Six months after EMR, a surveillance colonoscopy is performed, during which the endoscopic characteristics of the scar are determined by the endoscopist and biopsies of the scar are collected to determine residual or recurrent adenoma. In case of an aberrant scar morphology, multiple biopsies of the lesion are taken with a standard biopsy forceps. Lesions suspect for adenoma will be treated directly following the local protocol. The resected fragments will undergo histological analysis. Any other irregularities in and around the scar will be biopsied separately.

### Study end points

The primary outcome is the incidence of clinically significant DB, defined as anal blood loss occurring after the completion of the procedure necessitating emergency department consultation, blood transfusion, prolongation of hospital stay, re-hospitalization, or re-intervention (either repeat endoscopy, angiography or surgery) [5, 10, 20–23]. Self-limiting bleeding managed on an outpatient basis is not considered to be DB.

PC may improve patients’ health status, prevent serious complications of DB, reduce the demand for healthcare, and lower costs. The secondary outcomes therefore are (1) severity of DB (see Supplementary File 1 for definition of DB severity), (2) procedure time, (3) perforation rate, (4) EMR scar evaluation at the first surveillance colonoscopy after 6 months, (5) adenoma recurrence rate at 6 months, (6) health-related quality of life, and (7) direct and indirect costs related to PC.

### Sample size considerations

DB incidence after EMR in the right colon has been reported to range between 7 and 12.7% after EMR [6, 7]. Based on these studies, we believe that PC after standard EMR may be able to reduce delayed bleedings by 7.8% (from 9.8 to 2%) in a superiority design. With a 2-sided significance level of 5% (0.1% used as symmetric stopping boundaries during the interim-analysis, and 4.9% used as nominal significance level) and power of 80%, a total of 310 patients are required. With an estimated drop out of 15%, a total of 356 patients (2 × 178) are required to demonstrate this effect.

### Study withdrawal

Subjects can leave the study at any time for any reason without any consequences. The investigator can decide to withdraw a subject from the study for urgent medical reasons. There will be no replacement of individual subjects after withdrawal.

### Assignment of interventions

#### Randomization and treatment allocation

Randomization will take place in a 1:1 ratio after complete radical EMR without a clinically indicated intraprocedural clip placement, using a web-based randomization module (Castor EDC, Amsterdam, The Netherlands). Participants are stratified by center with random block sizes of 2, 4, and 6 per stratum. A time schedule for the trial can be found in Table 1.

**Table 1:** Schedule of enrolment, interventions, and assessments

	Study period						
	Enrolment	Allocation	Post-allocation				Close-out
Timepoint	Week − 1	EMR + 0	EMR+ 0	+ 1 month	+ 3 months	+ 6 months	+ 6 months
**Enrolment:**							
**Eligibility screen**	X						
**Informed consent**	X						
**Allocation**		X					
**Interventions:**							
***PC post EMR***			X				
***Questionnaires (EQ-5D*,** ***iMCQ*,** ***iPCQ)***			X	X	X	X	
***Control group without PC***			X				
**Assessments:**							
***Collect baseline and procedure variables***	X	X	X				
***Collect primary outcome variables***			X	X			
***Standard of care surveillance colonoscopy***						X	
***Collect secondary outcome variables***						X	X

#### Blinding

Patients will be blinded for treatment allocation whenever possible. However, patients undergoing EMR under conscious sedation cannot be blinded for treatment allocation. Whereas treating physicians cannot be blinded, the Outcome Adjudication Committee (OAC) evaluating the endpoints will be blinded for treatment allocation.

### Data collection and management

Coded study data will be collected in a web-based case record form (CRF) in Castor EDC. Patients will have the option to fill out the questionnaires with the end-to-end encrypted mobile “Improve” app (Open HealthHub, Utrecht, The Netherlands). After the data collection is complete, data are locked and saved for 15 years according to national law and regulations.

Missing data within a complete follow-up term will be prevented as much as possible. The reason for missing data will be reported. In case of > 5% non-selective missing data, multiple imputation may be performed. Sensitivity analyses will be performed with a different assumption for the distribution of the missing data than that was used in the primary analysis [24, 25].

### Statistical analysis methods

#### Primary analysis

Comparison of the primary endpoint DB will be performed according to intention-to-treat, using a mixed model regression analysis to correct for clustering of data, resulting in an estimated relative risk and 95% confidence intervals. Scale variables will be presented as mean ± standard deviation and in case of skewed distributions as median and range. Values will be compared by Student’s *T* test, Wilcoxon rank sum test, *χ*^2^ test, or Fisher exact test as appropriate. A two-tailed *p* value < 0.05 is considered statistically significant. A type 1 error will be controlled for by only taking one presentation of the primary endpoint (DB) per patient in the statistical analysis. In case of multiple presentations for bleeding, the highest gradation for severity will be included in the analysis. The outcomes of the intention-to-treat analysis will be compared to an exploratory per-protocol analysis.

In case of disbalances in baseline characteristics, potential confounders for DB will be determined with a *χ*^2^ test or unpaired *t* test where appropriate. Correction for potential confounders with a *p* ≤ 0.15 will be performed using multiple regression analysis. Considering the expected incidence of DB, the statistical power will allow to correct for two confounding factors. We expect at least anticoagulant medication to be a potential confounder for DB.

#### Analyses of secondary outcomes

The secondary endpoints will be compared between study groups using Student’ *T* test or *χ*^2^ test whenever appropriate. Lastly, a cost-effectiveness analysis will be performed. As a societal perspective allows for a more complete economic evaluation as compared to a health care perspective alone, three cost categories will be analyzed: direct medical costs, direct non-medical costs, and indirect costs (see Supplementary File 1 for a definition of these secondary endpoints).

#### Economic evaluation

All costs will be estimated in accordance with Dutch guidelines concerning cost-effectiveness studies [26, 27]. The price of clipping will be calculated based on the price of the Quick Clip Pro - Single Use Repositionable Clip (Olympus, Japan) in the Netherlands. Health care cost utilization and productivity losses will be estimated based on customized versions of the standardized questionnaires iMTA MCQ (Medical Cost Questionnaire) and iMTA PCQ (Productivity Cost Questionnaire). Loss of paid and unpaid work will be valued by means of the friction cost approach. Complete costs will be calculated for individual patients by multiplying actual health care resource use and unit prices [28, 29] and will be compared directly between study groups.

The impact of morbidity on the quality of life of patients will be assessed by the EQ-5D questionnaire. The individual scored items will be valued by the Dutch tariff. Health Utility Index scores will be used to derive a quality-adjusted life year (QALY) estimate for each patient [30, 31]. Incremental cost-effectiveness and cost-utility ratios will be calculated to reflect the extra costs per patient with poor outcome prevented and the extra costs per additional QALY respectively. Uncertainty in the cost-effectiveness ratio will be presented non-parametrically using bootstrap techniques. If correction for baseline disbalances is needed, regression techniques including bootstrapping will be used with the Net Monetary Benefit as outcome parameter. Results will be shown graphically by means of a cost-utility plane and cost-effectiveness acceptability curves with varying values of willingness-to-pay up to €80.000.

### Monitoring

#### Data monitoring

According to the Dutch Federation of Universities (NFU) standard for risk assessment and monitoring, the trial was graded as having a negligible risk for participants. Therefore, no Data Safety Monitoring Board or Data Monitoring Committee was indicated. Nonetheless, a blinded OAC comprising 3–4 independent gastroenterologists will assess and weigh all severe adverse events after completion of the trial and decide whether these concord with definitions of the study endpoints. Using primary source data and blinded for treatment allocation (if possible), each member of the committee will individually evaluate the disease course of each patient. Disagreements will be resolved at a plenary consensus meeting. Only after consensus has been reached on each individual endpoint, a final analysis will be performed.

#### Harms

All adverse events will be reported to the study coordinator regardless of a supposed relation to the trial intervention, who will in turn report all deaths and serious adverse events to the Central Committee on Research involving Human Subjects (CCMO) according to Dutch rules and legislation.

Being the primary outcome measure, DB was exempted of this reporting obligation. The relationship of all adverse events to the study intervention will be investigated by the primary investigators and the OAC. All adverse events will be followed until they have abated or until a stable situation has been reached.

#### Auditing

Based on the NFU standard for risk assessment and monitoring, each participating hospital will be visited by an independent monitor/auditor to audit adherence to the study protocol and to local research regulations. They will ensure correct handling of data and compare a random sample of the collected data to their source documents.

#### Interim analysis

An interim analysis of the primary endpoint will be performed when 50% of patients (*n* = 178) have been randomized and had 30 days of follow-up, aided by an independent statistician. The Haybittle-Peto approach is used to test efficacy, using symmetric boundaries at *p* < 0.001 to provide continuation or stopping advice. Finally, the steering committee will decide on the continuation of the trial.

## Discussion

The CLIPPER trial has been designed to determine whether PC is effective in preventing clinically significant DB after EMR of large proximal colorectal polyps. The current study design was improved by considering and discussing potential drawbacks of published or ongoing trials with the principal investigators. The following suggestions were made and implemented in the CLIPPER study design:

1. Randomization after EMR. In this way, non-radical EMRs are not included in the intervention arm and therapeutic clipping for perforation or intraprocedural bleeding can be performed to the discretion of the endoscopist without causing cross-over. The reasons for exclusion of a non-randomized patient after informed consent are registered [32].
2. Inclusion of patients with only one polyp, instead of multiple, enables one to estimate the pure effect of PC on DB and its severity [32].
3. Inclusion of only right sided polyps, which are shown to be associated with a higher DB rate and significant decrease of DB after PC [32, 33].
4. Inclusion of polyps sized 20–60 mm. Previous studies have shown no effect of PC for smaller polyps [34–36], and polyps over 60 mm were excluded in view of feasibility of complete closure [33].
5. Cost-effectiveness analysis. Some studies have predicted cost-effectiveness of PC based on economic models [37, 38]. We will analyze cost-effectiveness based on healthcare utility questionnaires and clinical data.
6. Follow-up period of 6 months. Previous studies have reported that clips can affect scar formation and cause clip artifacts that can be hard to distinguish from recurrent polyp tissue [14]. In order to determine these late clip effects, we included the first surveillance colonoscopy 6 months post-EMR in the follow-up period.

A potential drawback of this study is caused by the timing of the informed consent. In Dutch daily clinical practice, most polyps in the lower range of these inclusion criteria, especially the 2–3 cm polyps, will already be removed by EMR in the same session. As it would neither be ethical nor feasible to approach all colonoscopy patients for informed consent, we will likely include more 3–6 cm lesions undergoing repeat colonoscopy for EMR than 2–3 cm lesions. This needs to be taken into account when interpreting our results.

In conclusion, the CLIPPER trial is powered and designed to determine the efficacy and effectiveness of PC in everyday practice, in order to fill the gaps in our understanding of the place of PC in the endoscopy unit.

## Trial status

The first patient was randomized on May 15, 2018. To date, 19 hospitals are open for inclusion and 217 patients have been randomized. The inclusion is slightly below schedule, which is at least partly due to the recent COVID-19 outbreak. Protocol version 2.2 is being used and was approved on January 23, 2018, with an amendment approved on August 23, 2018. Patient recruitment is expected to last until mid-2021.

## Supplementary Information

**Additional file 1.** Definitions of Secondary Endpoints. *Severity of DB was defined according to the ASGE working party document for adverse events in colonoscopy [20].

**Additional file 2.** List of parameters collected in the Case Record Form.

**Additional file 3.** SPIRIT Checklist.

## Data Availability

The datasets generated and/or analyzed during the current study are available after publication from the primary investigator (EvG) on reasonable request.

## Abbreviations

EMR: Endoscopic mucosal resection
PC: Prophylactic clipping
DB: Delayed bleeding
NCCS: National Colorectal Cancer Screening Program
iFOBT: Immunological fecal occult blood testing
NBI: Narrow band imaging
APC: Argon plasma coagulation
ASA: American Society of Anesthesiology grade
LMWH: Low-molecular-weight heparin
DOAC: Direct oral anticoagulants
PI: Principle investigator
CRF: Case record form
QALY: Quality-adjusted life year
OAC: Outcome Adjudication Committee
(S)AE: (Severe) adverse event
METC: “Medisch Ethische Toetsings Commissie” (English: Medical Ethical Review Board)
CCMO: Central Committee on Research involving Human Subjects
NNT: Number needed to treat
ARD: Absolute risk difference

## References

[1] http://www.nationaalkompas.nl/gezondheid-en-ziekte/ziekten-en-aandoeningen/kanker/dikkedarmkanker/bereik-effecten/ [Available from: http://www.nationaalkompas.nl/gezondheid-en-ziekte/ziekten-en-aandoeningen/kanker/dikkedarmkanker/bereik-effecten/. Last accessed Dec 2017.

[2] Veldhuizen-Eshuis H, Van CM, Delden JA, Van GL, Hoebee B, Lock AJJ. Uitvoeringstoets bevolkingsonderzoek naar darmkanker: Opsporing van darmkanker in praktijk gebracht. RIVM Rapport 225101003. 2011. Available from: https://www.rivm.nl/publicaties/uitvoeringstoets-bevolkingsonderzoek-naar-darmkanker-opsporing-van-darmkanker-in.

[3] Bourke M (2011). Endoscopic mucosal resection in the colon: a practical guide. Tech Gastrointest Endosc.

[4] Buddingh KT, Herngreen T, Haringsma J, van der Zwet WC, Vleggaar FP, Breumelhof R (2011). Location in the right hemi-colon is an independent risk factor for delayed post-polypectomy hemorrhage: a multi-center case-control study. Am J Gastroenterol.

[5] Burgess NG, Metz AJ, Williams SJ, Singh R, Tam W, Hourigan LF (2014). Risk factors for intraprocedural and clinically significant delayed bleeding after wide-field endoscopic mucosal resection of large colonic lesions. Clin Gastroenterol Hepatol.

[6] Bahin FF, Naidoo M, Williams SJ, Hourigan LF, Ormonde DG, Raftopoulos SC (2015). Prophylactic endoscopic coagulation to prevent bleeding after wide-field endoscopic mucosal resection of large sessile colon polyps. Clin Gastroenterol Hepatol.

[7] Lee CK, Lee SH, Park JY, Lee TH, Chung IK, Park SH (2009). Prophylactic argon plasma coagulation ablation does not decrease delayed postpolypectomy bleeding. Gastrointest Endosc.

[8] Fujihara S, Mori H, Kobara H, Nishiyama N, Kobayashi M, Rafiq K (2013). The efficacy and safety of prophylactic closure for a large mucosal defect after colorectal endoscopic submucosal dissection. Oncol Rep.

[9] Matsumoto M, Fukunaga S, Saito Y, Matsuda T, Nakajima T, Sakamoto T (2012). Risk factors for delayed bleeding after endoscopic resection for large colorectal tumors. Jpn J Clin Oncol.

[10] Liaquat H, Rohn E, Rex DK (2013). Prophylactic clip closure reduced the risk of delayed postpolypectomy hemorrhage: experience in 277 clipped large sessile or flat colorectal lesions and 247 control lesions. Gastrointest Endosc.

[11] Dokoshi T, Fujiya M, Tanaka K, Sakatani A, Inaba Y, Ueno N (2015). A randomized study on the effectiveness of prophylactic clipping during endoscopic resection of colon polyps for the prevention of delayed bleeding. Biomed Res Int.

[12] Osada T, Sakamoto N, Ritsuno H, Murakami T, Ueyama H, Matsumoto K (2016). Closure with clips to accelerate healing of mucosal defects caused by colorectal endoscopic submucosal dissection. Surg Endosc.

[13] Gupta S, Lieberman D, Anderson JC, Burke CA, Dominitz JA, Kaltenbach T (2020). Recommendations for follow-up after colonoscopy and polypectomy: a consensus update by the US Multi-Society Task Force on Colorectal Cancer. Am J Gastroenterol.

[14] Pellise M, Desomer L, Burgess NG, Williams SJ, Sonson R, McLeod D (2016). The influence of clips on scars after EMR: clip artifact. Gastrointest Endosc.

[15] Sreepati G, Vemulapalli KC, Rex DK (2015). Clip artifact after closure of large colorectal EMR sites: incidence and recognition. Gastrointest Endosc.

[16] Chan AW, Tetzlaff JM, Altman DG, Laupacis A, Gotzsche PC, Krleza-Jeric K (2013). SPIRIT 2013 statement: defining standard protocol items for clinical trials. Ann Intern Med.

[17] Chan AW, Tetzlaff JM, Gotzsche PC, Altman DG, Mann H, Berlin JA (2013). SPIRIT 2013 explanation and elaboration: guidance for protocols of clinical trials. BMJ.

[18] Brouwer MA, van Hooft JE, Meijer K, Mundt MW, Peters FTM, Prins AJR, et al. Nederlandse Richtlijn Beleid antitrombotische therapie rondom endoscopische procedures. Nederlandse Vereniging van Maag-Darm-Leverartsen. 2016. Available from: https://www.mdl.nl/sites/www.mdl.nl/files/richlijnen/Richtlijn_antitrombotische_therapie_final_mei_2016.pdf.

[19] Parra-Blanco A, Kaminaga N, Kojima T, Endo Y, Uragami N, Okawa N (2000). Hemoclipping for postpolypectomy and postbiopsy colonic bleeding. Gastrointest Endosc.

[20] Cotton PB, Eisen GM, Aabakken L, Baron TH, Hutter MM, Jacobson BC (2010). A lexicon for endoscopic adverse events: report of an ASGE workshop. Gastrointest Endosc.

[21] Lee HS, Park JJ, Kim SU, Lee JE, Leem GL, Kim Y (2016). Incidence and risk factors of delayed postpolypectomy bleeding in patients with chronic liver disease. Scand J Gastroenterol.

[22] Burgess NG, Williams SJ, Hourigan LF, Brown GJ, Zanati SA, Singh R (2014). A management algorithm based on delayed bleeding after wide-field endoscopic mucosal resection of large colonic lesions. Clin Gastroenterol Hepatol.

[23] Bahin FF, Rasouli KN, Williams SJ, Lee EY, Bourke MJ (2016). Prophylactic clipping for the prevention of bleeding following wide-field endoscopic mucosal resection of laterally spreading colorectal lesions: an economic modeling study. Endoscopy..

[24] White IR, Horton NJ, Carpenter J, Pocock SJ (2011). Strategy for intention to treat analysis in randomised trials with missing outcome data. BMJ.

[25] Ibrahim JG, Chu H, Chen MH (2012). Missing data in clinical studies: issues and methods. J Clin Oncol.

[26] Ligtenberg mdF, Staal mmPC, Goettsch dWG, Knies mdS. Kosteneffectiviteit in de zorg: Op weg naar een genuanceerd en geaccepteerd gebruik van kosteneffectiviteitsgegevens in de zorg. College voor Zorgverzekeringen; 2013. Report No.: 2013082521 Contract No.: 2013082521. Available from: https://www.zorginstituutnederland.nl/publicaties/rapport/2013/09/30/kosteneffectiviteit-in-de-zorg.

[27] prof. dr. M.J. IJzerman, dr. M.J. Al, prof. dr. A. de Boer, prof. dr. W.B.F. Brouwer, prof. dr. J.J. van Busschbach, dr. M.G.W. Dijkgraaf, et al. Richtlijn voor het uitvoeren van economische evaluaties in de gezondheidszorg. In: Nederland Z, editor. 2016.

[28] Oostenbrink JB, Buijs-Van der Woude T, van Agthoven M, Koopmanschap MA, Rutten FF (2003). Unit costs of inpatient hospital days. Pharmacoeconomics..

[29] Oostenbrink JB, Koopmanschap MA, Rutten FFH (2002). Standardisation of costs. Pharmacoeconomics..

[30] Lamers LM, Stalmeier PF, McDonnell J, Krabbe PF, van Busschbach JJ (2005). Measuring the quality of life in economic evaluations: the Dutch EQ-5D tariff. Ned Tijdschr Geneeskd.

[31] Dolan P (1997). Modeling valuations for EuroQol health states. Med Care.

[32] Pohl H, Grimm IS, Moyer MT, Hasan MK, Pleskow D, Elmunzer BJ, et al. Clip closure prevents bleeding after endoscopic resection of large colon polyps in a randomized trial. Gastroenterology. 2019;157(4):977-984.e3. 10.1053/j.gastro.2019.03.019. 10.1053/j.gastro.2019.03.019 PMC8224988 30885778

[33] Albeniz E, Alvarez MA, Espinos JC, Nogales O, Guarner C, Alonso P (2019). Clip closure after resection of large colorectal lesions with substantial risk of bleeding. Gastroenterology.

[34] Feagins LA, Nguyen AD, Iqbal R, Spechler SJ (2014). The prophylactic placement of hemoclips to prevent delayed post-polypectomy bleeding: an unnecessary practice? A case control study. Dig Dis Sci.

[35] Matsumoto M, Kato M, Oba K, Abiko S, Tsuda M, Miyamoto S (2016). Multicenter randomized controlled study to assess the effect of prophylactic clipping on post-polypectomy delayed bleeding. Dig Endosc.

[36] Shioji K, Suzuki Y, Kobayashi M, Nakamura A, Azumaya M, Takeuchi M (2003). Prophylactic clip application does not decrease delayed bleeding after colonoscopic polypectomy. Gastrointest Endosc.

[37] Albeniz E, Gonzalez MF, Martinez-Ares D, Alonso P, Guarner-Argente C, Mugica F (2016). Cost-effectiveness of prophylactic clipping after colorectal endoscopic mucosal resection and economic impact according to a bleeding risk score. Gastrointest Endosc.

[38] Shah ED, Pohl H, Rex DK, Morales SJ, Feagins LA, Law R. Routine prophylactic clip closure is cost saving after endoscopic resection of large colon polyps in a Medicare population. Gastroenterology. 2020;158(4):1164-1166.e3. 10.1053/j.gastro.2019.11.015. 10.1053/j.gastro.2019.11.015 31738918

