# Supplementary material for: Clip placement to prevent delayed bleeding after colonic endoscopic mucosal resection (CLIPPER): study protocol for a randomized controlled trial
Source: Trials. 2021 Jan 18;22:63. doi: 10.1186/s13063-020-04996-7 (PMC7813164; doi:10.1186/s13063-020-04996-7)
Supplement: Supplementary file 1 — Additional file 1. Definitions of Secondary Endpoints. *Severity of DB was defined according to the ASGE working party document for adverse events in colonoscopy [20]. [file 13063_2020_4996_MOESM1_ESM.docx]

***Supplementary File 1: Definitions of Secondary Endpoints***

| **Severity of DB*** | |
| --- | --- |
| Mild | any unplanned hospital admission, or prolongation of hospital stays for 3 days or fewer |
| Moderate | unplanned hospital admission of 4 to 10 days, transfusion, repeat endoscopy, any interventional radiology procedure, or intensive care unit (ICU) admission for 1 night |
| Severe | admission to the ICU for more than 1 night, unplanned admission for more than 10 nights, surgery, or permanent disability |
| **Cost effectiveness** | |
| Direct Medical Costs | Costs that are generated by healthcare utilization and include hospital admission, therapeutic and diagnostic procedures, medication, and visits to primary and paramedical healthcare providers. |
| Direct non-medical costs | Costs that reflect utilization of non-healthcare resources generated by the disease, e.g. travel to and from health care providers, private household assistance, over-the-counter medication, etc. |
| Indirect Costs | Costs that are associated with loss of productivity due to impaired ability to work. |

**Severity of DB was defined according to the ASGE working party document for adverse events in colonoscopy.^14^*
