# Supplementary material for: Clip placement to prevent delayed bleeding after colonic endoscopic mucosal resection (CLIPPER): study protocol for a randomized controlled trial
Source: Trials. 2021 Jan 18;22:63. doi: 10.1186/s13063-020-04996-7 (PMC7813164; doi:10.1186/s13063-020-04996-7)
Supplement: Supplementary file 2 — Additional file 2. List of parameters collected in the Case Record Form. [file 13063_2020_4996_MOESM2_ESM.docx]

***Supplementary file 2: List of parameters collected in the CRF***

**Baseline**

- Patient number (coded)
- Randomization
- Gender
- Age
- Height and weight to calculate BMI
- Chronic hypertension (yes/no)
- Anticoagulant drug use and stop-time
- Smoking behavior
- Alcohol drinking behavior
- Family history

**Local EMR protocol**

- Endoscope brand and type (i.e.: brand of scopes: Fuji/Olympus/Pentax, type: pediatric colonoscope or normal colonoscope)
- Type and brand of snares
- Type of injection fluid (with or without adrenaline)
- Piecemeal or En bloc removal
- Amount of snare-coagulations (tempi/pieces) to remove the polyp.
- Sedation
- Means of cleaning of the colon
- Post-operative stay
- Soft-coagulation/ APC of the resection margins (yes/no)
- Treatment of intra-procedural bleeding (snare tip / forceps coagulation)

**During EMR (reported in colonoscopy report)**

- Experience level of the endoscopist
  - Low: <50 EMR life time
  - Moderate: 50-150 EMR life time OR >150 lifetime and <50 EMR/year
  - High: >150 EMR life time with an average case load of 50/year OR >350 EMR’s lifetime
- Blood pressure at endoscopy
- Boston Bowel Preparation Scale
- Morphologic polyp type according to Paris classification
- Kato classification for degree of lifting
- Kudo classification for pit pattern
- Polyp size in mm
- Polyp location in cm ab ano
- Photos of the polyp prior to resection
- Procedure time in minutes (total procedure time and time for clip(s) placement)
- Description of used material: type of scope/snare etc. (see section 3)
- Description of procedure and method (e.g. injection-, cap-, or ligation-assisted; coagulation of margins, etc.)
- Number of used clips
- Occurrence of immediate intra-procedural bleeding
- Management of immediate intra-procedural bleeding (i.e. APC)
- Photos of the resected area (directly after EMR)
- In case of clipping: photos of the clipped area
- Estimation of closure
- A photo is made of the closed EMR resection-site. The endoscopist will estimate the:
- Percentage of wound closure
- Grade of PC closure:
  - Successful: at least 1 clip every 0,5-1cm.
  - Partial successful: 1 clips every >1cm, and/or evidently opening edges of the borders of the EMR wound.
  - Failure: <50% of clips placed
- Clip placement failure:
  - Rate: Amount of successfully placed clips versus total amount of clips used.
  - Reason: detachment, wrong placement, technical failure, etc.
- Placement of tattoo for localization at follow up.

**30-day follow up/on re-admission:**

- Onset of DB in days after polypectomy
- Occurrence of Postpolypectomy Syndrome
- Perforation rate
- Histology of the resected polyp
- Depth of invasion
- Differentiation grade
- ER presentation
- Hospital readmission (yes/no)
- Length of hospital and intensive care unit (ICU) stay
- Treatment of DB (type and number of interventions (colonoscopy, surgery, angiography with or without coiling)
- Blood transfusion

**180 day follow up:**

- EMR site evaluation (clips versus no clips) with photos and biopsy.
  - Clipping scar (clip artifact)
  - Adenoma recurrence
  - Doubt about the diagnosis
- Where possible the images will be taken with the Pentax Zoomscope with 136° enlargement.
- Direct medical and non-medical costs, and indirect costs.
